# Multigenomics Reveals the Causal Effect of Herpes Simplex Virus in Alzheimer’s Disease: A Two-Sample Mendelian Randomization Study

**DOI:** 10.3389/fgene.2021.773725

**Published:** 2022-01-05

**Authors:** Yuwei Zhang, Jiaojiao Qu, Li Luo, Zhongshun Xu, Xiao Zou

**Affiliations:** ^1^ College of Life Sciences, Institute of Fungal Resources, Guizhou University, Guiyang, China; ^2^ Key Laboratory of Plant Resource Conservation and Germplasm Innovation in Mountainous Region (Ministry of Education), Institute of Agro-Bioengineering, Guizhou University, Guiyang, China; ^3^ College of Tea Sciences, Guizhou University, Guiyang, China

**Keywords:** HSV, genome, Mendelian randomization, Alzheimer’s disease, causality

## Abstract

In recent years, the herpes virus infectious hypothesis for Alzheimer’s disease (AD) has gained support from an increasing number of researchers. Herpes simplex virus (HSV) is a potential risk factor associated with AD. This study assessed whether HSV has a causal relationship with AD using a two-sample Mendelian randomization analysis model. Six single-nucleotide polymorphisms (SNPs) associated with HSV-1 and thirteen SNPs associated with HSV-2 were used as instrumental variables in the MR analysis. We estimated MR values of relevance between exposure and the risk of AD using inverse-variance weighted (IVW) method, MR-Egger regression (Egger), and weighted median estimator (WME). To make the conclusion more robust and reliable, sensitivity analyses and RadialMR were performed to evaluate the pleiotropy and heterogeneity. We found that anti-HSV-1 IgG measurements were not associated with risk of AD (OR, 0.96; 95% CI, 0.79–1.18; *p* = 0.736), and the same was true for HSV-2 (OR, 1.03; 95% CI, 0.94–1.12; *p* = 0.533). The findings indicated that any HSV infection does not appear to be a genetically valid target of intervention in AD.

## 1 Introduction

Alzheimer’s disease (AD) is a complex chronic progressive degenerative disorder of the central nervous system, affecting primarily the elderly, which severely reduces the quality of life (Calabrò et al., 2021). According to the 2015 World Alzheimer Report, the number of AD patients is expected to double every 20 years, reaching up to 131.5 million by 2050 (Prince et al., 2015; Du et al., 2018) with the incidence rate of AD increasing exponentially after 65 years of age (Hou et al., 2019). AD is diagnosed after age 65 as late-onset AD (LOAD) and before age 65 as early-onset AD (EOAD). LOAD accounts for about 95% of AD cases. EOAD is essentially an inherited disease, with a 92%–100% heritability. In contrast, there are multiple factors influencing LOAD, which are sporadic (Laval and Enquist, 2021). AD has two central pathological features: the extracellular deposition of amyloid plaques and intracellular accumulation of neurofibrillary tangles (NFTs). Amyloid plaques are mainly composed of amyloid-β (Aβ) protein and NFTs are composed of hyperphosphorylated tau proteins. Hence, there have been contrasting theories proposed about the underlying pathogenesis of AD, such as amyloid cascade hypothesis, Tau protein hypothesis, and oxidative stress. Nonetheless, to date, current therapies have failed to delay disease progression. In recent years, the herpes virus infection hypothesis has received a renewed interest by scientists who believe that infection is the main cause of AD.

In the 1980s, herpes simplex virus (HSV) was first proposed to be associated with AD after viral genetic material was discovered in the human brain as well as virus-induced lesions present in the limbic system were associated with AD (Ball, 1982). The viruses belong to the *Alphaherpesviridae* subfamily of the Herpesviridae family, including HSV-1 and HSV-2, which are ubiquitous human pathogens (Piret and Boivin, 2020). Previous studies (Wozniak et al., 2010) found that HSV-1 DNA was present in the brains of both AD patients and normal elderly people; however, in the brains of AD patients, HSV-1 DNA was found within 90% of the plaques and 72% of HSV-1 DNA was associated with plaques, while in the brains of normal elderly people, only 24% of HSV-1 DNA was associated with plaques. Thus, it was proposed that the HSV-1 infects infants and remains latent in the peripheral nervous system. Reactivation of latent HSV-1 infections may cause local neuronal damage and inflammation, which over time may lead to the deposition of Aβ and abnormal phosphorylation of tau in the brain. A recent study proposed that Aβ deposition and abnormal phosphorylation of tau were the brain’s immune response to HSV-1 (Eimer et al., 2018). However, another recent study showed that AD associated β-amyloid does not protect against HSV-1 infection in the mouse brain (Bocharova et al., 2021).

To date, the precise molecular events, and biological pathways underlying the disease have yet to be identified and the existing evidence does not definitively support the herpesviruses hypothesis of AD. The deposition of Aβ and abnormal phosphorylation of tau are not necessarily the cause of AD, but may be the result of other risk factors leading to AD. Meanwhile, given the existence of unmeasured confounding variables and reverse causation, previous epidemiological studies have demonstrated a correlation but no direct causal relationship between HSV and AD, which allows for a re-evaluation of the theory as a possible strategy.

Multi-omics research probes the interaction between multiple factors in biological systems, including genomics, epigenomics, transcriptomics, proteomics, metabolomics, and microbiomics. These factors jointly affect phenotypes and physiological traits. With the development of high-throughput sequencing technology, omics research continues to provide more extensive data. Through high-throughput sequencing, omics, and data integration studies, we can comprehensively and systematically understand the relationship between various factors in the fields of basic research, molecular biology, clinical diagnosis, and drug discovery. (Hasin et al., 2017).

Genomics is the earliest discipline stemming from histology, and focuses on the study of the entire genome, and is currently the most established discipline in the field. Genomics focuses on the identification of genetic variants associated with disease, treatment response, or patient prognosis (Hasin et al., 2017). With the successful development of next-generation sequencing (NGS) technology and the completion of the human genome project and the International Human Genome HapMap project (HapMap), genome-wide association studies (GWAS) have become a method for identifying millions of genetic variants related to complex diseases (GWAS catalog https://www.ebi.ac.uk/gwas/home) in different human populations. In such studies, millions of individuals are genotyped for many genetic markers, and the genotypes and phenotypes are subjected to statistical analysis at a population level. Significant differences in minor allele frequencies (MAF) between cases and controls are thought to be markers affecting the trait. GWAS studies provide an invaluable contribution to our understanding of complex phenotypes (Hasin et al., 2017).

Mendelian randomization (MR) is a strategy for evaluating the causality of risk factors of a disease using genetic variants from the GWAS as instrumental variables (IV) (Lawlor et al., 2008). It is based on the Mendelian inheritance law of “random allocation of parental alleles to offspring” in meiosis, which is equivalent to a randomized controlled trial using genotypes. MR analysis can remove the limitations of traditional epidemiology. As alleles were randomly allocated at conception, confounders cannot influence the result of the allocated alleles. Because the disease cannot alter genetic variants, reverse causation may be avoided.

**FIGURE 1:**
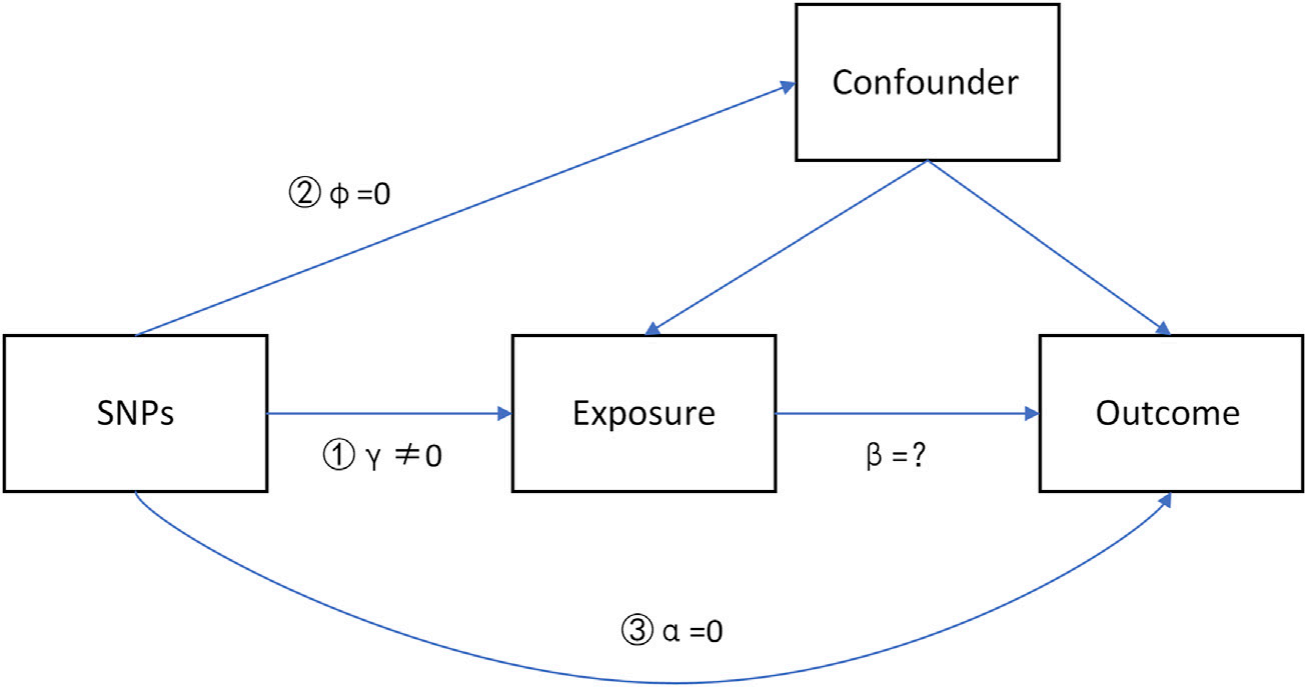
Directed acyclic graph (DAG) model of instrumental variables in causal associations.

IVs should satisfy three major hypotheses (Figure 1), which have been widely described in recent studies (Liu et al., 2018; Liu et al., 2021).

1) The IV is associated with the exposure (γ≠0, strong IVs).
2) The IV is not associated with the confounders (*φ* = 0).
3) The IV does not influence the outcome through some pathways other than the exposure (*α* = 0, no directional pleiotropy).

Scepanovic et al. (2018) measured quantitative IgG responses to HSV-1 and HSV-2 infection in humoral immunity to explore the influence of genetic factors on the variability of humoral responses. After genome-wide genotyping of single-nucleotide polymorphisms (SNPs) and imputation, they examined associations between genetic variants and HSV-1 and HSV-2 IgG and performed two genome-wide association analyses. The International Genomics of Alzheimer’s Project Consortium (IGAP) (Lambert et al., 2013) conducted a meta-analysis using genotyped and imputed data on four previously published GWAS datasets and obtained a novel genome-wide association analysis demonstrating the relationship of genetic variants with AD. In the present study, we used many SNPs of multi-genome association analysis as IVs to perform two-sample MR analysis (Gibran et al., 2018).

## 2 Materials and Methods

### 2.1 Data Sources

The exposure risk factors considered in this study were HSV-1 and HSV-2. The genetic variations for both exposures were anti-HSV-1 IgG measurement and anti-HSV-2 IgG measurement, which were downloaded from a GWAS study of Scepanovic et al. (2018), which was the basis of the summary data published in the NHGRI-EBI GWAS (https://www.ebi.ac.uk/gwas). The sample was derived from The French Milieu Interieur cohort, which was stratified by sex (500 men, 500 women) and age (200 individuals from each decade of life, between 20 and 70 years of age). The HSV-2 datasets contained 208 cases and 792 controls, and HSV-1 datasets contained 645 cases and 355 controls.

The summary data of AD derived from the International Genomics of Alzheimer’s Project Consortium (IGAP), which was a sizeable two-stage research based on GWASs of AD in 74,046 diseased and normal individuals of European ancestry (Lambert et al., 2013). In stage 1, the IGAP performed a meta-analysis of four previously published GWAS datasets containing 17,008 AD patients and 37,154 controls, using genotyped and imputed data on 7,055,881 SNPs. The outcome data from IGAP stage 1 results were from the study of Kunkle et al. (2019). Table 1 shows the detailed descriptions of IGAP stage 1 data.

**TABLE 1:** Description of consortium datasets for IGAP stage 1.

Consortium	*N* (cases/controls)	Percent women (cases/controls)	Case mean AAO (s.d.)	Control mean AAE (s.d.)
ADGC	10,273/10,892	59.4/58.6	74.7 (7.7)	76.3 (8.1)
CHARGE	1,315/12,968	63.6/57.8	82.7 (6.8)	72.8 (8.6)
EADI	2,243/6,017	64.9/60.7	68.5 (8.9)	74.0 (5.4)
GERAD	3,177/7,277	64.0/51.8	73.0 (8.5)	51.0 (11.8)
*N*	17,008/37,154			

AAO, age at onset; AAE, age at examination.

### 2.2 Methods

All the analyses were performed using R version 4.1.0 software.

#### 2.2.1 Selection of Instrumental Variables

The most critical step in MR design is to identify suitable genetic variants as IVs. First, we extracted SNPs that had significant (*p* < 1 × 10^–5^) associations with HSV-1 and HSV-2. Then, we performed a linkage disequilibrium (LD) analysis to exclude mutual linkage SNPs and to discard non-biallelic SNPs. LD (*r* ^2^ < 0.001, kb > 10,000) was applied to select IVs of HSV-1 and HSV-2. The samples used to estimate the LD effect derived from individuals of European ancestry from the 1,000 Genome Project. Correlated SNPs in LD were excluded using the “clump_data” function of the “TwoSampleMR” R package. As a result, 7 SNPs were identified for HSV-1 and 13 SNPs for HSV-2.

#### 2.2.2 Harmonize

A summary set can generate errors if the effect alleles for the SNP effects in the exposure and outcome datasets are different. We aligned the effect alleles for exposure and outcome based on reported effect alleles and effect allele frequencies using the “harmonise_data” function of the “TwoSampleMR” R package (Gibran et al., 2018). Furthermore, we used *F*-statistics (Bowden et al., 2016) to measure the strength of the selected IVs. If the *F*-statistic was more than ten, genetic variants were generally deemed to be a strong IV.

#### 2.2.3 Mendelian Randomization

We conducted the MR analysis using inverse-variance weighted (IVW) regression analysis, MR-Egger regression analysis, and weighted median estimator (WME). IVW can provide accurate estimates when the IV satisfies the MR assumptions that there are no invalid IVs (Burgess et al., 2013). The mean effect estimate of IVW is derived from a random effect IVW meta-analysis of the Wald ratios (SNP-outcome associations divided by SNP-exposure associations) estimated for each IV (Staley and Burgess, 2017). MR-Egger regression is robust for invalid instruments, and can be used to test for directional pleiotropy, providing an estimate of the causal effect adjusted for a variable’presence. In MR-Egger, an intercept that differs from zero estimates the average pleiotropy effect across the genetic variants, which indicates that the IVW estimate is biased (Bowden et al., 2015). However, MR-Egger regression is more easily influenced by regression dilution, so that it should be approximated using the *I* ^2^ statistic. If *I* ^2^ is high (*I* ^2^ > 0.9), Egger regression can be considered an unbiased estimation (Bowden et al., 2016). The WME provides a consistent, valid estimate if at least half of the IVs are valid (Verbanck et al., 2018). MR analyses were performed using the R-based package “TwoSampleMR”.

#### 2.2.4 Sensitivity Analysis

The three methods described above were applied to analyze causal estimation, and we performed the following additional analyses and assessments to examine the robustness of the results. First, we used Egger intercept to test the pleiotropy of SNPs (Burgess and Thompson, 2017). Then, we calculated the heterogeneity among SNPs using Cochran’s *Q*-statistic to assess the robustness of IVs (Kippersluis and Rietveld, 2017). Furthermore, to evaluate whether the MR estimate was driven or biased by a single SNP that might have an enormous pleiotropic effect, RadialMR was applied to present a more straightforward detection of outliers and to correct horizontal pleiotropy by removing outliers (Bowden et al., 2018). All sensitivity analyses were performed using the R-based package “TwoSampleMR” and “RadialMR”.

## 3 Results and Discussion

### 3.1 The Causality of HSV-1 and AD

After removing the palindrome SNP (rs1738233), six SNPs for HSV-1 infection were identified, which were significant (*p* < 1 × 10^–5^) and independent (*r* ^2^ < 0.001). The *F*-statistics for the six SNPs were all more than 10, which indicated that all six IVs were strong instruments (Table 2).

**TABLE 2:** SNPs significantly (*p*-value < 1 × 10^−5^) and independently (*r* ^2^ < 0.001) associated with herpes simplex virus type 1 (HSV-1) infection (SNPs = 6).

Exposure	SNP	Effect allele	Other allele	EAF	Beta	SE	*p*-value	*F*-statistic
HSV-1	rs10977313	T	G	0.11	−0.12	0.02	2.97 × 10^−7^	26.9
	rs1446553	A	G	0.25	0.07	0.02	9.85 × 10^−6^	19.9
	rs34018815	A	G	0.10	−0.11	0.02	8.35 × 10^−6^	20.2
	rs58599785	T	C	0.16	0.08	0.02	4.91 × 10^−6^	21.3
	rs78421079	T	C	0.08	−0.12	0.03	6.49 × 10^−6^	20.7
	rs8020017	G	A	0.40	−0.06	0.01	9.85 × 10^−6^	19.9

**FIGURE 2:**
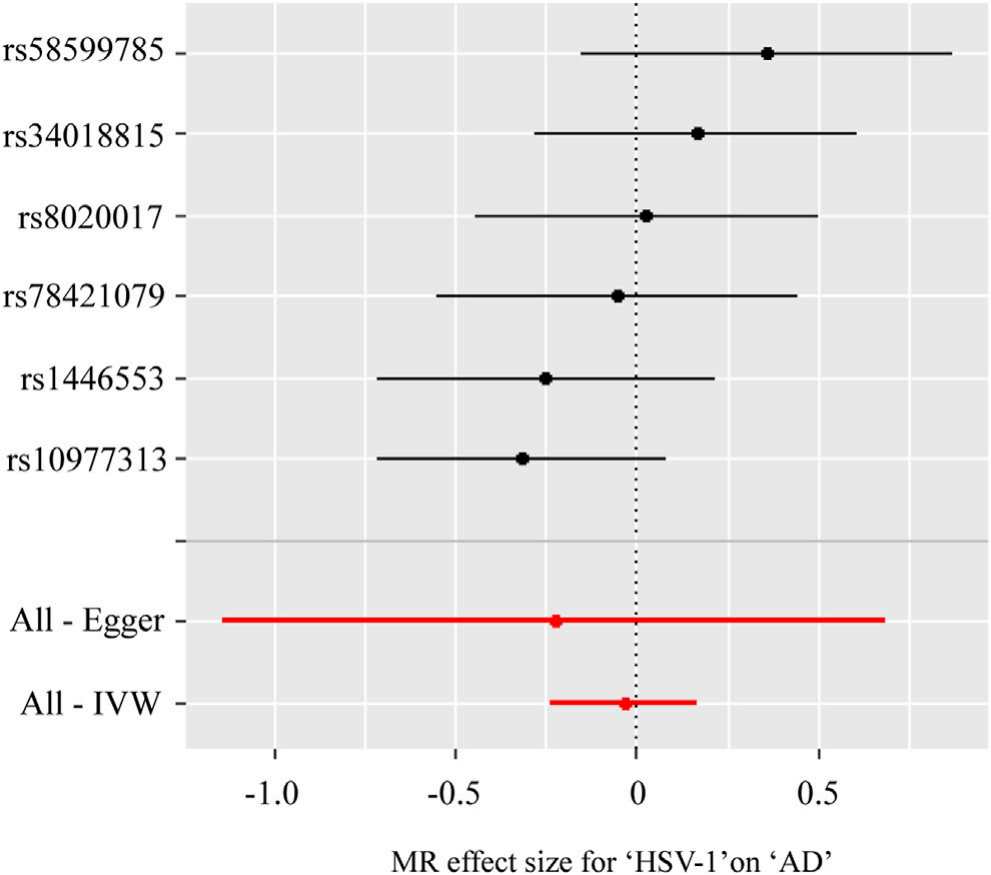
Forest plot for two-sample Mendelian randomization effect size between Herpes simplex virus type 1 (HSV-1) and Alzheimer’s disease (AD).

Table 3; Figure 2 showed the estimated associations of HSV-1 risk factor with AD from MR analysis. Genetically predicted HSV-1 infection was not associated with AD risk using IVW (OR = 0.96, *p* = 0.736), WME (OR = 0.97, *p* = 0.833), and MR-Egger (OR = 0.79, *p* = 0.653). The MR-Egger intercept indicated no directional pleiotropy (intercept = 0.018, *p* = 0.694), suggesting that horizontal pleiotropy was unlikely to influence the IVW estimate. The *I* ^2^ statistics was 0.958, indicating that relative bias did not materially affect the standard MR-Egger analysis. Cochran’s *Q* test showed no existence of heterogeneity of SNPs (Cochran’s *Q*-statistic = 5.83, *p* = 0.322), while RadialMR showed that there were no outliers in the six SNPs.

**TABLE 3:** Association of six SNPs for HSV-1 infection with AD using MR with different methods.

Outcome	SNPs		OR	95% CI	*p*-value	Pleiotropy	Heterogeneity		*I* ^2^
						Intercept *p*-value	Cochran’s *Q*-statistic	*p*-value	
AD	6	IVW	0.96	[0.79–1.18]	0.736		5.836	0.323	
		WME	0.97	[0.77–1.23]	0.833				
		MR-Egger	0.79	[0.32–1.99]	0.653	0.694	5.586	0.232	0.958

### 3.2 The Causality of HSV-2 and AD

Thirteen SNPs for HSV-2 infection were identified, which were both significant (*p* < 1 × 10^–5^) and independent (*r* ^2^ < 0.001) (Table 4). The *F*-statistics for the thirteen SNPs were more than 10, which indicated that they were strong IVs.

**TABLE 4:** SNPs significantly (*p*-value < 1 × 10^−5^) and independently (*r* ^2^ < 0.001) associated with herpes simplex virus type 2 (HSV-2) infection (SNPs = 13).

Exposure	SNP	Effect allele	Other allele	EAF	Beta	SE	*p*-value	*F*-statistic
HSV-2	rs10888851	G	C	0.11	−0.24	0.05	3.27 × 10^−6^	23.2
	rs10782620	G	T	0.39	0.16	0.03	2.60 × 10^−6^	23.7
	rs12042287	C	T	0.27	−0.16	0.03	7.67 × 10^−6^	21.3
	rs10174926	C	T	0.13	−0.24	0.04	9.72 × 10^−7^	25.9
	rs72804080	G	A	0.13	0.26	0.05	1.92 × 10^−7^	29.5
	rs355547	C	T	0.39	0.17	0.04	2.00 × 10^−6^	24.3
	rs35213774	G	A	0.11	0.26	0.05	1.10 × 10^−6^	25.6
	rs6826994	A	G	0.06	−0.30	0.06	6.76 × 10^−6^	21.6
	rs113043839	A	G	0.06	0.32	0.07	6.94 × 10^−6^	21.5
	rs10100854	T	C	0.37	−0.15	0.03	7.00 × 10^−6^	21.5
	rs10964023	T	G	0.19	−0.19	0.04	3.58 × 10^−6^	23.0
	rs17802723	G	C	0.08	−0.28	0.06	7.90 × 10^−6^	21.3
	rs10790877	A	G	0.47	−0.16	0.03	7.82 × 10^−7^	26.4

**FIGURE 3:**
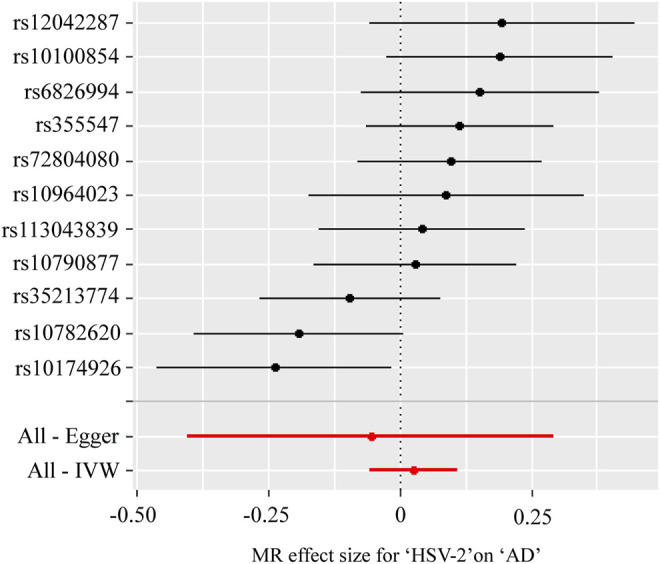
Forest plot for two-sample Mendelian randomization effect size between Herpes simplex virus type 2 (HSV-2) and Alzheimer’s disease (AD).

Table 5; Figure 3 showed the estimated associations of HSV-2 risk factors with AD from the MR analysis. Genetically predicted HSV-2 infection was not associated with the AD using IVW (OR = 1.03, *p* = 0.533), WME (OR = 1.08, *p* = 0.121), and MR-Egger (OR = 0.95, *p* = 0.764). The MR-Egger intercept indicated that there was no directional pleiotropy (intercept = 0.017, *p* = 0.646). Furthermore, the Cochran’s *Q*-statistic indicated the existence of heterogeneity of SNPs (Cochran’s *Q*-statistic = 18.8, *p* = 0.04). Meanwhile, no outliers were detected using RadialMR.

**TABLE 5:** Association of thirteen SNPs for HSV-2 infection with AD using MR with different methods.

Outcome	SNPs		OR	95% CI	*p*-value	Pleiotropy	Heterogeneity		*I* ^2^
						Intercept *p*-value	Cochran’s *Q*-statistic	*p*-value	
AD	13	IVW	1.03	[0.94–1.12]	0.533		18.802	0.043	
		WME	1.08	[0.98–1.18]	0.121				
		MR-Egger	0.95	[0.67–1.34]	0.764	0.017	18.343	0.031	0.959

## 4 Discussion

We found that both HSV-1 and HSV-2 were not causally associated with an increased risk of AD using genetic variation as instrumental variables. Kwok and Schooling (2021) used the GWAS summary statistics data from the French Milieu Interieur cohort, the United Kingdom biobank, and the US 23 and Me Study, pointing out that HSV-1 and HSV-2 were not associated with AD. SY et al. (2021) used the GWAS summary statistics data from the 23 and Me cohort, indicating the same result.

### 4.1 The Result of HSV and AD

Although the causality of the association is unclear, many studies have proven that HSV is not unrelated to AD. HSV-1 virus was detected in the brains of both AD patients and elderly normal people. However, most of the AD patients were APOE-ε4 gene carriers. The herpesvirus hypothesis proposes that HSV-1 enters the brains of APOE-ε4 carriers, where it remains a latent life with limited transcription and low protein synthesis. In response to immunosuppression, peripheral infection, and inflammation, HSV-1 reactivates, creating a combination of viral action and inflammatory effects that are poorly repaired by APOE-ε4 carriers, ultimately leading to the development of AD (Itzhaki, 2018). In addition, a recent study pointed out novel molecular mechanisms through which recurrent HSV-1 infection may affect neuronal aging, likely contributing to neurodegeneration (Napoletani et al., 2021).

We inferred that our results may have occurred mainly due to several reasons. The major reason is that reactivation after latent HSV-1 infection may be responsible for a pathogenetic mechanism of AD, and IgM is a marker of activation of primary infection. Our study used anti-HSV-1 IgG antibodies rather than IgM as a proxy for HSV-1 infection, implicitly demonstrating that previous HSV-1 infection is not associated with AD risk. Another reason is the speculation that HSV-1 infection is not a risk factor for cognitive decline but rather a phenomenon that co-occurs with neuro-inflammation or as a result of neuro-inflammation.

Meanwhile, we found that HSV-2 was not causally associated with an increased risk of AD using genetic variation as an instrumental variable. This is probably because that according to the available epidemiological observations, HSV-2 mainly invades the genitalia and the area from the waist down and is not associated with the brain.

Future studies should perform MR analyses using anti-HSV-1 IgM antibodies as an IV for HSV-1 infection. What we can conclude, however, is that AD is not simply a single factor disease caused by HSV, but that it encompasses complex disease mechanisms.

### 4.2 Advantages and Challenges of MR Analysis

In the investigation of risk factors for AD, traditional research methods present many challenges in discovering the cause of the disease. Observational studies can only demonstrate a correlation rather than causality between exposure and outcome due to confounding factors and reverse causality. Cohort studies can make causal arguments but waste time. Random control trials (RCT) are considered the gold standard for clinical diagnosis and have a solid causal view. However, when applied by researchers, they are difficult to practice due to medical ethics and the many limitations of the design process. For these reasons, MR analysis has become a more convenient and effective way of exploring the causal links between risk factors and AD.

The application of MR analysis in this study has several advantages. First, reverse causality can be avoided, and second, it can prevent the interference of confounding factors. MR analysis can also address situations where an intervention experiment cannot be performed because of ethical restrictions (Zheng et al., 2017). Our exposure data were obtained from a publicly available GWAS database published with credibility. Our outcome data derived from a study conducted by the IGAP with a large sample population.

Nonetheless, our study also has some limitations. First, our data samples were based on individuals of European ancestry, so the results are not representative of all races. Second, the sample size of the exposure data was not sufficiently large, leading to the low power of statistics and false negatives. However, a significant number of IVs can lead to high power but inevitable heterogeneity and pleiotropy of IVs. This is where the general challenge of MR.

## 5 Conclusion

We implemented a two-sample MR to demonstrate the causal relationship between HSV infection and AD risk. The SNPs were independent and strong instrumental variables, and the result was robust and reliable. Our findings indicated the negative association between any HSV IgG and AD. Further research is needed to investigate whether HSV IgM is corelated with AD, and whether HSV infections that co-occur with neuro-inflammation are more relevant.

## Data Availability

Publicly available datasets were analyzed in this study. The AD data can be found here: https://www.niagads.org/datasets/ng00075. The HSV data can be found here: https://www.ebi.ac.uk/gwas/efotraits/EFO_0009349 and https://www.ebi.ac.uk/gwas/efotraits/EFO_0009350.

## Abbreviations

AD, Alzheimer’s disease; APOE-ε4, apolipoprotein e gene; CI, confidence interval; GWAS, genome-wide association studies; HSV, herpes simplex virus; IGAP, International Genomics of Alzheimer’s Project; IV, instrumental variables; IVW, inverse-variance weighted; LD, linkage disequilibrium; MR, Mendelian randomization; OR, odds ratio; WME, weighted median estimator.

## References

[B1] Ball M. J. (1982). "Limbic Predilection in Alzheimer Dementia: Is Reactivated Herpesvirus Involved?". Can. J. Neurol. Sci. 9, 303–306. 10.1017/s0317167100044115 7116237

[B2] Bocharova O. Pandit N. P. Molesworth K. Fisher A. Mychko O. Makarava N. (2021). Alzheimer's Disease-Associated β-amyloid Does Not Protect against Herpes Simplex Virus 1 Infection in the Mouse Brain. J. Biol. Chem. 297, 100845. 10.1016/j.jbc.2021.100845 34052228 PMC8214219

[B3] Bowden J. Spiller W. Del Greco M F. Sheehan N. Thompson J. Minelli C. (2018). Improving the Visualization, Interpretation and Analysis of Two-Sample Summary Data Mendelian Randomization via the Radial Plot and Radial Regression. Int. J. Epidemiol. 47, 2100. 10.1093/ije/dyy265 30423109 PMC6280936

[B4] Bowden J. Davey Smith G. Burgess S. (2015). Mendelian Randomization with Invalid Instruments: Effect Estimation and Bias Detection through Egger Regression. Int. J. Epidemiol. 44, 512–525. 10.1093/ije/dyv080 26050253 PMC4469799

[B5] Bowden J. Del Greco M. F. Minelli C. Davey Smith G. Sheehan N. A. Thompson J. R. (2016). Assessing the Suitability of Summary Data for Two-Sample Mendelian Randomization Analyses Using MR-Egger Regression: The Role of the I2 Statistic. Int. J. Epidemiol. 45, dyw220–1974. 10.1093/ije/dyw220 PMC5446088 27616674

[B6] Burgess S. Butterworth A. Thompson S. G. (2013). Mendelian Randomization Analysis with Multiple Genetic Variants Using Summarized Data. Genet. Epidemiol. 37, 658–665. 10.1002/gepi.21758 24114802 PMC4377079

[B7] Burgess S. Thompson S. G. (2017). Interpreting Findings from Mendelian Randomization Using the MR-Egger Method. Eur. J. Epidemiol. 32, 377–389. 10.1007/s10654-017-0255-x 28527048 PMC5506233

[B8] Calabrò M. Rinaldi C. Rinaldi C. Santoro G. Crisafulli C. (2021). The Biological Pathways of Alzheimer Disease: A Review. AIMS Neurosci. 8, 86–132. 10.3934/Neuroscience.2021005 33490374 PMC7815481

[B9] Du X. Wang X. Geng M. (2018). Alzheimer's Disease Hypothesis and Related Therapies. Transl Neurodegener 7, 2. 10.1186/s40035-018-0107-y 29423193 PMC5789526

[B10] Eimer W. A. Vijaya Kumar D. K. Navalpur Shanmugam N. K. Rodriguez A. S. Mitchell T. Washicosky K. J. (2018). Alzheimer's Disease-Associated β-Amyloid Is Rapidly Seeded by Herpesviridae to Protect against Brain Infection. Neuron 100, 1527–1532. 10.1016/j.neuron.2018.11.043 30571943

[B11] Gibran H. Zheng J. Benjamin E. Wade K. H. Valeriia H. Baird D. (2018). The MR-Base Platform Supports Systematic Causal Inference across the Human Phenome. ELife 7, e34408. 29846171 10.7554/eLife.34408 PMC5976434

[B12] Hasin Y. Seldin M. Lusis A. (2017). Multi-omics Approaches to Disease. Genome Biol. 18, 83. 10.1186/s13059-017-1215-1 28476144 PMC5418815

[B13] Hou Y. Dan X. Babbar M. Wei Y. Hasselbalch S. G. Croteau D. L. (2019). Ageing as a Risk Factor for Neurodegenerative Disease. Nat. Rev. Neurol. 15, 565–581. 10.1038/s41582-019-0244-7 31501588

[B14] Huang S.-Y. Yang Y.-X. Kuo K. Li H.-Q. Shen X.-N. Chen S.-D. (2021). Herpesvirus Infections and Alzheimer's Disease: A Mendelian Randomization Study. Alz Res. Ther. 13, 158. 10.1186/s13195-021-00905-5 PMC8464096 34560893

[B15] Itzhaki R. F. (2018). Corroboration of a Major Role for Herpes Simplex Virus Type 1 in Alzheimer's Disease. Front. Aging Neurosci. 10, 324. 10.3389/fnagi.2018.00324 30405395 PMC6202583

[B16] Kunkle B. W. Grenier-Boley B. Sims R. Bis J. C. Damotte V. Naj A. C. (2019). Author Correction: Genetic Meta-Analysis of Diagnosed Alzheimer's Disease Identifies New Risk Loci and Implicates Aβ, Tau, Immunity and Lipid Processing. Nat. Genet. 51, 1423–1424. 10.1038/s41588-019-0358-210.1038/s41588-019-0495-7 31417202 PMC7265117

[B17] Kwok M. K. Schooling C. M. (2021). Herpes Simplex Virus and Alzheimer's Disease: A Mendelian Randomization Study. Neurobiol. Aging 99, e11–101. 10.1016/j.neurobiolaging.2020.09.025 33139072

[B18] Lambert J. C. Ibrahim-Verbaas C. A. Harold D. Naj A. C. Sims R. Bellenguez C. (2013). Meta-analysis of 74,046 Individuals Identifies 11 New Susceptibility Loci for Alzheimer's Disease. Nat. Genet. 45, 1452–1458. 10.1038/ng.2802 24162737 PMC3896259

[B19] Laval K. Enquist L. W. (2021). The Potential Role of Herpes Simplex Virus Type 1 and Neuroinflammation in the Pathogenesis of Alzheimer's Disease. Front. Neurol. 12, 458. 10.3389/fneur.2021.658695 PMC8055853 33889129

[B20] Lawlor D. A. Harbord R. M. Sterne J. A. C. Timpson N. Davey Smith G. (2008). Mendelian Randomization: Using Genes as Instruments for Making Causal Inferences in Epidemiology. Statist. Med. 27, 1133–1163. 10.1002/sim.3034 17886233

[B30] Liu G. Zhoa Y. Jin S. Hu Y. Wang T. Tian R. (2018). Circulating Vitamin E Levels and Alzheimer's Disease: A Mendelian Randomization Study. Neurobiol. Aging 72, 181–189. 10.1016/j.neurobiolaging.2018.08.008 30174138

[B31] Liu H. Zhang Y. Zhang H. Wang L. Wang T. Han Z. (2021). Effect of Plasma Vitamin C Levels on Parkinson's Disease and Age at Onset: A Mendelian Randomization Study. J. Transl. Med. 19, 221. 10.1186/s12967-021-02892-5 34030714 PMC8142636

[B21] Napoletani G. Protto V. Marcocci M. E. Nencioni L. Palamara A. T. De Chiara G. (2021). Recurrent Herpes Simplex Virus Type 1 (HSV-1) Infection Modulates Neuronal Aging marks in *In Vitro* and *In Vivo* Models. Ijms 22, 6279. 10.3390/ijms22126279 34208020 PMC8230621

[B22] Piret J. Boivin G. (2020). Immunomodulatory Strategies in Herpes Simplex Virus Encephalitis. Clin. Microbiol. Rev. 33. 10.1128/CMR.00105-19 PMC7018500 32051176

[B23] Prince M. Wimo A. Guerchet M. Ali G.-C. Wu Y.-T. Prima M. (2015). World Alzheimer Report 2015:the Global Impact of Dementia: An Analysis of Prevalence, Incidence, Cost and Trends. London, United Kingdom: Alzheimer's Disease International, 84. Available at: https://kclpure.kcl.ac.uk/portal/en/publications/world-alzheimer-report-2015-the-global-impact-of-dementia(ae525fda-1938-4892-8daa-a2222a672254)/export.html .

[B24] Scepanovic P. Alanio C. Alanio C. Hammer C. Hodel F. Bergstedt J. (2018). Human Genetic Variants and Age Are the Strongest Predictors of Humoral Immune Responses to Common Pathogens and Vaccines. Genome Med. 10, 59. 10.1186/s13073-018-0568-8 30053915 PMC6063007

[B25] Staley J. R. Burgess S. (2017). Semiparametric Methods for Estimation of a Nonlinear Exposure-Outcome Relationship Using Instrumental Variables with Application to Mendelian Randomization. Genet. Epidemiol. 41, 341–352. 10.1002/gepi.22041 28317167 PMC5400068

[B26] van Kippersluis H. Rietveld C. A. (2017). Pleiotropy-robust Mendelian Randomization. Int. J. Epidemiol. 47, 1279–1288. 10.1093/ije/dyx002 PMC6124631 28338774

[B27] Verbanck M. Chen C.-Y. Neale B. Do R. (2018). Detection of Widespread Horizontal Pleiotropy in Causal Relationships Inferred from Mendelian Randomization between Complex Traits and Diseases. Nat. Genet. 50, 693–698. 10.1038/s41588-018-0099-7 29686387 PMC6083837

[B28] Wozniak M. A. Mee A. P. Itzhaki R. F. (2010). Herpes Simplex Virus Type 1 DNA Is Located within Alzheimer's Disease Amyloid Plaques. J. Pathol. 217, 131–138. 10.1002/path.2449 18973185

[B29] Zheng J. Baird D. Borges M.-C. Bowden J. Hemani G. Haycock P. (2017). Recent Developments in Mendelian Randomization Studies. Curr. Epidemiol. Rep. 4, 330–345. 10.1007/s40471-017-0128-6 29226067 PMC5711966

